# Upgraded D22 SEC–SANS setup dedicated to the biology community

**DOI:** 10.1107/S1600576723004119

**Published:** 2023-06-12

**Authors:** Anne Martel, Cristina Cocho, Francesca Caporaletti, Mark Jacques, Abdelali El Aazzouzi, Franck Lapeyre, Lionel Porcar

**Affiliations:** a Institut Laue–Langevin, 71 avenue de Martyrs, Grenoble 38042, France; b Department of Physics, Chemistry and Biology (IFM), Linköping University, Linköping, Sweden; University of Sydney, Australia

**Keywords:** size-exclusion chromatography, SEC, small-angle neutron scattering, SANS, SEC–SANS

## Abstract

Described here is the current status of the upgraded *in situ* size-exclusion chromatography (SEC) system implemented with the D22 small-angle neutron scattering (SANS) instrument at the Institut Laue–Langevin. Since its initial proof of principle in 2016, this SEC–SANS arrangement has been continuously requested by the user community, leading to the design of an upgraded version. A detailed description of the setup and its control is provided, and a few examples of protein structural investigations are presented, which will highlight the various possibilities and limitations of the setup to optimize experimental success.

## Abstract 2

This publication describes the stable version of a liquid chromatography system combined with the small-angle neutron scattering (SANS) instrument D22 at Institut Laue–Langevin to run size-exclusion chromatography (SEC) immediately before SANS measurement.

## 1. Introduction

Small-angle scattering (SAS) is a highly effective technique for determining the low-resolution structure of biological macromolecules in solution. However, it is also highly sensitive to the presence of aggregates. To ensure that the sample is monodisperse, it is recommended to subject it to size-exclusion chromatography (SEC) immediately prior to SAS measurement.

SEC filters the sample through a gel that separates biomolecules on the basis of their size. The gel contains polymer beads with pores of different sizes, and biomolecules passing through a column of the gel under pressure are separated according to the size of the pore they can enter. The column is first equilibrated with the desired elution buffer and then the sample is injected. Biomolecules larger than the biggest pore will not be slowed on the column by entering the gel beads and are thus eluted first, followed by molecules in order of decreasing molecular weight across the column separation range. Finally, very small molecules and the sample solvent are eluted after flowing through the whole column volume of buffer. SEC is a crucial step in protein purification to ensure optimal aggregation-free sample quality and to avoid mis­interpretation of data.

Nowadays, SEC is routinely combined with small-angle X-ray scattering (SAXS) on many synchrotron biological SAXS beamlines (Pérez & Vachette, 2017 ▸; Brennich *et al.*, 2017 ▸; Graewert *et al.*, 2020 ▸) at facilities such as Diamond (B21, https://www.diamond.ac.uk/Instruments/Soft-Condensed-Matter/small-angle/B21/description.html#), APS (BioCAT, https://www.bio.aps.anl.gov/pages/about-saxs.html), SSRL (BL4-2, https://www-ssrl.slac.stanford.edu/smb-saxs/content/documentation/sec-saxs) and ALS (SIBYLS, https://sibyls.als.lbl.gov/htsaxs/instructions/secsaxs), as well as on benchtop instruments (Bucciarelli *et al.*, 2018 ▸; Inoue *et al.*, 2019 ▸).

While SEC–SAXS has become widespread on high-brilliance synchrotron sources, small-angle neutron scattering (SANS) applied to biological macromolecules (bioSANS) has remained in its infancy, historically requiring much longer exposure times (several hours) due to the lower flux and brilliance of neutron beams. As a result, only extremely stable samples could be analysed, and a direct combination of SEC on neutron instruments did not make sense.

Over time, instruments and sample quality have improved significantly, and thanks to the high neutron flux now available at the Institut Laue–Langevin (ILL), exposure times have decreased significantly to routinely less than 10 min. This breakthrough has opened the possibility of measuring less stable samples and thus led to interest in combining a SEC setup that filters the sample directly before SANS measurement.

The first prototype and proof of principle of the combination of SEC and SANS measurements was published in 2016 (Jordan *et al.*, 2016 ▸), along with examples of the benefits it brings. Since these pioneering measurements, other structural biology studies have benefited from the SEC–SANS combination (Johansen *et al.*, 2018 ▸), guiding the development of a fully automated and dedicated optimized setup. This development has now achieved stability and this publication aims to describe it in detail, including its limitations, to help future users successfully plan their experiments.

## 2. Modular hardware

### 2.1. Global view

The development of the SEC–SANS system on the ILL SANS instrument D22 was guided by several requirements.

(i) Obtaining neutron beamtime is notoriously difficult, particularly for bioSANS that requires a high flux of neutrons. To use the beamtime as efficiently as possible, the system was designed with two columns, one in equilibration while the other is in use for sample elution and measurement.

(ii) Unlike X-rays, neutrons do not induce radiation damage in the samples, which means that the samples can be reused for quality control and further analysis. To facilitate this, a fraction collector was included in the setup.

(iii) An automated injection system, and the ability to choose amongst a set of elution buffers, were required to plan several elution/measurement runs one after the other.

(iv) Since biological samples are often temperature sensitive, a proper temperature control system is indispensable.

(v) The measurement cell must be wide enough to use the largest possible neutron beam to optimize the neutron flux, but at the same time it should not lead to the simultaneous exposure of different species eluting close to each other. A compromise between SANS signal and chromatography resolution is needed.

(vi) For proper normalization of the SANS data by mol­ecule concentration, the sample absorbance must be measured. For accuracy, this is measured on exactly the same sample volume as the neutron beam. Simultaneous recording of chromatograms at different wavelengths is desirable to study biomolecular complexes.

(vii) Direct control of the SEC system by *NOMAD*, the D22 control software (Mutti *et al.*, 2011 ▸), was highly desirable in order to synchronize the SEC process and SANS exposures, and to integrate chromatograms in NeXus SANS data files. This was also a requirement to provide the best support to users in case of problems.

**Figure 1:**
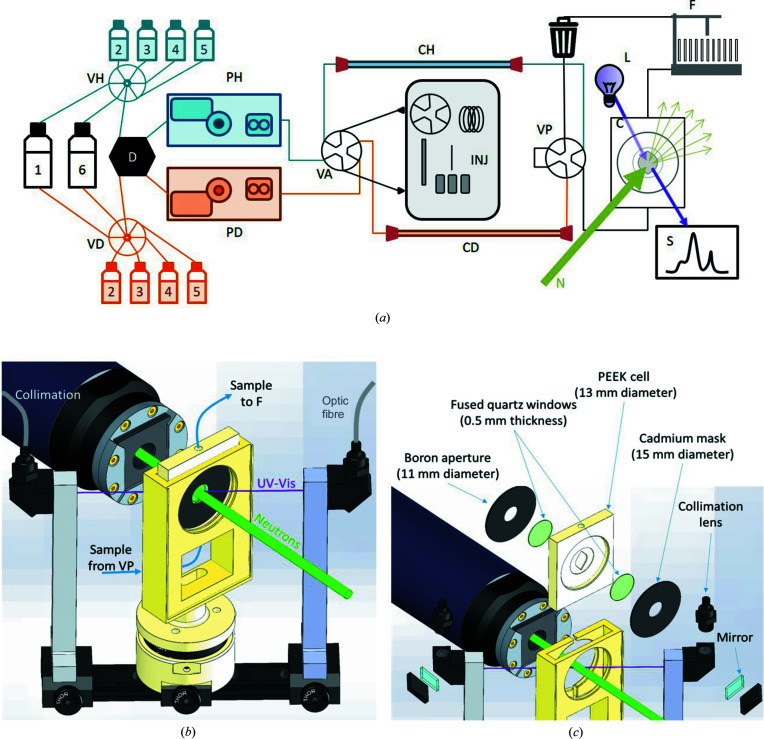
(*a*) A schematic diagram of the SEC–SANS system, including pumps H and D (PH and PD), valves H and D (VH and VD), columns H and D (CH and CD), the degasser (D), the lamp (L), the spectrophotometer (S), the cell (C), valves ANTE and POST (VA and VP), the fraction collector (F), and the sample injector (INJ). (*b*) A technical drawing of the cell as installed in the SEC–SANS setup. (*c*) An exploded view of the same cell, with details of the windows, aperture, mask and optic fibre connections. The green line represents the neutron beam and the purple line represents the UV–visible light beam.

Meeting all these constraints has resulted in a modular and rather complex setup. Subsequently, each component had to be integrated into a user-friendly and intuitive interface. We have purchased SEC equipment from Knauer, a brand that provides us with computer control protocols. Each piece of equipment is connected to the D22 control server via ethernet cables, except for the sample injector (Alias, RS232), which unfortunately does not come equipped with an RJ45 port. Fig. 1 ▸(*a*) provides a schematic diagram of the fluidic connections, Fig. 1 ▸(*b*) shows a technical drawing of the cell as it is mounted in the setup and Fig. 1 ▸(*c*) shows an exploded view, detailing the various elements along the beam path. Each individual component is described below.

### 2.2. Pumps, columns and buffer selection valves

The two columns installed in the setup are designated H and D, and each one is connected to a pump and a buffer valve, also labelled H or D. The pumps utilized are Blue Shadow 40P pumps (Knauer), capable of withstanding up to 30 bar (1 bar = 100 000 Pa) of pressure and equipped with 10 ml ceramic heads that can deliver flow rates ranging from 10 µl to 10 ml per minute. The H and D valves are six-port and six-way Azura valves (Knauer) that select the buffer to be pumped, with up to four different buffers available per pump. By default, positions 1 and 6 are connected to water and 20% ethanol, respectively, for column cleaning. A degasser is placed between the valve and the pumps to degas the chosen buffer before it enters the column.

Superdex 200 and 75 Increase (10/300 or 5/150) columns (Cityvia) are typically used in the setup, but users are encouraged to bring their own columns if they require a specific type of separation or are concerned about contamination. Although less resolutive, the 5/150 versions of these columns dilute the sample much less than their 10/300 counterparts, resulting in more realistic requirements for the initial sample concentration. Specifically, the 5/150 dilutes the sample by a factor of two, while the 10/300 dilutes it by a factor of seven. The 10/300 columns must be used for detergent exchange, whereas the 5/150 columns are preferred for buffer exchange.

### 2.3. Automation/sample injector

To program several consecutive SEC–SANS runs, it is necessary to automate sample loading and column switching via *NOMAD*. The Alias automatic sample injector (Spark Holland) can handle up to 96 samples in septum-closed vials of 2 ml. Vial inserts are typically used to inject sample volumes of 50 to 250 µl. The injector syringe has a capacity of 250 µl, but larger volumes can be injected by repeated plunger movements. The injector needle is automatically cleaned (inside and outside) with 20% ethanol between injections.

Two Azura valves (Knauer) are used to switch between the two columns: valve ANTE is located before the columns and valve POST is located after the columns. Both valves are six-port and two-way. Valve ANTE selects the column on which the sample will be injected, while valve POST selects the column from which the eluent is sent to the SANS measurement cell and fraction collector. Together, these valves automate the switch between the two columns.

### 2.4. Sample cell

The sample cell used in this study was designed and fabricated in house [see Figs. 1 ▸(*b*) and 1 ▸(*c*)]. It is made of PEEK (polyether ether ketone), a chemically inert and rigid material that can be accurately machined. The bottom of the cell is connected to valve POST, while the top is connected to the fraction collector using standard liquid chromatography connectors (10-32 threading). The sample flows upwards through 0.5 mm diameter channels that become funnel shaped as they reach the circular sample chamber. The cell windows consist of 0.5 mm thick UV coverslips (UQG Optics, part No. CFS-2050) with a diameter of 20 mm. The cell is sandwiched between a boron carbide beam-defining aperture and a cadmium mask with a 15 mm diameter hole.

**Figure 2:**
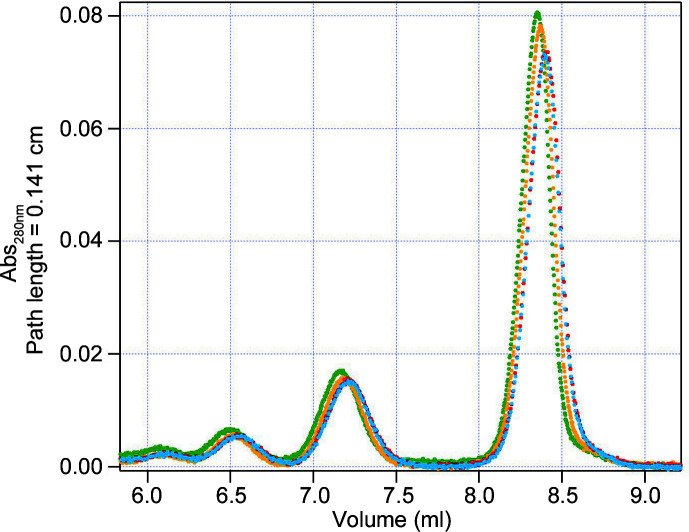
Chromatograms obtained from eluting BSA (5 mg ml^−1^) through a Superdex 200 10/300 Increase column, followed by measurement cells with either 13 mm (red and yellow) or 10 mm (blue and green) diameter, at two different flow rates: 0.2 ml min^−1^ (red and blue) and 0.1 ml min^−1^ (yellow and green).

To optimize the cell geometry and preserve the column separation resolution while using a large neutron beam to ensure a sufficient flux, two cell-aperture geometries were compared: a small one with a cell chamber diameter of 10 mm and an aperture diameter of 8.4 mm, and a large one with a cell chamber diameter of 13 mm and an aperture diameter of 11 mm. The comparison was performed using commercial bovine serum albumin (A4503-50G lyophilized powder, >96% purity, Sigma–Aldrich) simply diluted to 5 mg ml^−1^ in 50 m*M* Tris, 150 m*M* NaCl pH 7.5, without any further purification, at room temperature. Two elution flow rates were tested (0.1 and 0.2 ml min^−1^) with a constant flow rate throughout the elution, and the UV absorbance values at 280 nm were collected. Fig. 2 ▸ shows the four very similar chromatograms. The FWHM of the monomer peak (around 8.3 ml elution volume) varied from 0.202 ml for the small cell to 0.207 ml for the large cell at 0.1 ml min^−1^, and from 0.225 to 0.224 ml at 0.2 ml min^−1^. The cell diameter seems to have a negligible effect on sample dilution compared with the flow rate. In SANS measurements, using a 13 mm diameter cell and 11 mm diameter beam should provide a 2.4-fold increase in neutron flux compared with using a 10 mm diameter cell and 8.4 mm diameter beam. Therefore, the 13 mm diameter cell chamber with an 11 mm diameter aperture was chosen for user operation.

Besides the geometry, there are noticeable advantages to this cell:

(i) Its high transmission rate (98% of the incoming beam at a wavelength of 6 Å ± 10%).

(ii) Optimization of the sample volume actually used: 95 µl of sample are exposed out of a total cell volume of 140 µl, while for the usual Hellma cuvettes (100-1-40), filling a cell requires 180 µl of sample but only 70 µl are actually exposed.

(iii) The proximity of the boron carbide beam-defining aperture and cadmium mask, which avoids air scattering and improves signal/noise.

(iv) The flow-through geometry, which enables recording of the buffer signal under the same conditions as the protein signal for very accurate buffer subtraction.

### 2.5. Fraction collector

The Foxy R1 collector (Teledyne ISCO) can collect fractions in various formats such as 5 ml tubes, 2 ml tubes or 96-well plates of different volumes. The user can set the fraction size either in time (in seconds) or in volume (in the number of drops). Fraction collection is optional and can start at the first collector position or continue after the previous collection, enabling the user to append several SEC runs without any intervention.

### 2.6. *In situ* UV–visible absorbance

UV absorbance is measured using an Ocean HDX spectrophotometer (OceanOptics), through the same aperture as SANS measurement. This compact diode array spectrophotometer records one spectrum every 2 s during elution. Although the parameters can be adjusted, these spectra are typically the average of 20–50 spectra with 50–100 ms of exposure. A deuterium/halogen lamp (DH-2000, OceanOptics) serves as the light source. Both the lamp and the spectrophotometer are connected to the sample cell through 300 µm diameter optic fibres, collimation lenses and a 45° angle mirror (all purchased from OceanOptics/Idil fibre). The UV–visible light passes through the 1 mm thick sample cell at a 45° angle, resulting in a path length of 1.41 mm, and is detected as *I* ^UV^. The electronic noise intensity spectrum ( 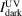 ) is measured and subtracted from all subsequent spectra. The buffer intensity spectrum 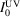 is measured at the beginning of each chromatography run. The absorbance spectrum is calculated from the intensity spectrum *I* ^UV^ as

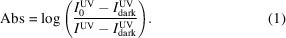




Both the absorbance and intensity spectra are recorded and saved in NeXus SANS data files, along with the chromatograms at four selected wavelengths. Note that the absorbance values are normalized by the exposure time but not by the sample path length (1.41 mm).

To maintain temperature control during experiments, the entire setup is enclosed in a large polyethylene insulated box. The box is equipped with three cooling units (Peltier thermo­electric air cooler, TE Technology) and connected to a Thermojet unit (SP Scientific). This setup allows for measurements to be taken at temperatures as low as 283 K without experiencing any water condensation issues.

## 3. Versatile control

*NOMAD*, the ILL instrument control software, provides a user-friendly interface that allows the user to select preset commands (or a set of commands), enter key variable parameters, and send them to the instrument or sample environment. By combining *NOMAD*’s flexibility with the hardware modularity of the SEC setup, it is possible to control each piece of equipment independently. To improve user comfort, we have also developed an integrated controller.

### 3.1. The modular running option

In the ‘Execution’ tab of *NOMAD*, users can program and send commands sequentially or in parallel to individual pieces of equipment in the SEC setup. The position of each valve and the flow rate of each pump can be set individually, and injections can be triggered with adjustable parameters for injected volume and sample vial position. The spectrophotometer can also be triggered to record chromatograms with selected parameters, including wavelength and duration, and a reference intensity spectrum ( 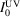 as a function of wavelength) is systematically recorded at the beginning of each chromatogram. Additionally, the fraction collector can be started and stopped according to preset parameters.

While the modularity of this system provides great flexibility, its complexity can make it difficult to handle. With numerous tunable parameters, setting each piece of equipment individually for each SEC run can be stressful and error prone for users. To address this issue, an integrated controller has been developed for user comfort and ease of use.

### 3.2. The integrated controller

**Figure 3:**
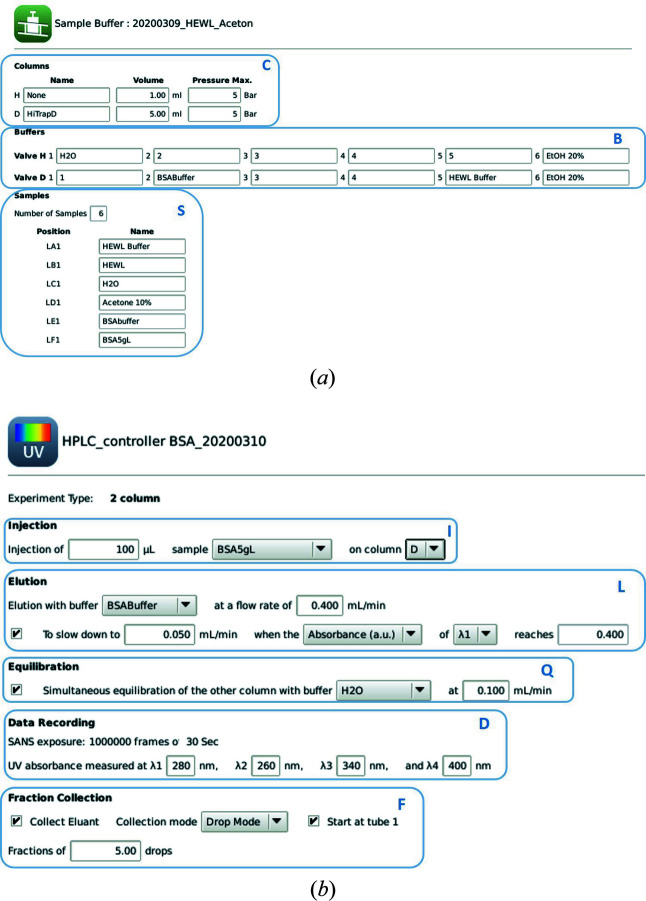
(*a*) The sample–buffer description table and (*b*) the SEC–SANS controller in the *NOMAD* Settings tab, enabling users to describe and save SEC elution runs by filling in a series of fields outlined in blue: C for columns, B for buffers, S for samples, I for injection, L for elution, Q for equilibration, D for data recording and F for fraction collection. Once these fields are defined, the SEC runs can be programmed one after another in the Execution tab.

The integrated controlller requires completion of two forms in the ‘Settings’ tab, as presented in Fig. 3 ▸. The first of these is the ‘sample buffer’ definition form [Fig. 3 ▸(*a*)], which enables the user to set up the specific chromatography configuration for their experiment through the three fields outlined in blue:

Field C describes the two columns the user is going to use (designated H and D), with their volume and maximum pressure.

Field B lists the compositions and positions (1 to 6, on the H or D valve) of the elution buffers. Note that buffers H can be used for elution on column H and buffers D on column D. The same buffer bottle can be connected to a port H and a port D.

Field S lists the names and positions of samples placed in the injector (L or R, for left and right tray, positions A to F and 1 to 8 in the tray). Although this option has not been used up to now, the interface also includes the option to work with a 2 mm thick sample cell.

The second form is the ‘HPLC controller’ tab, shown in Fig. 3 ▸(*b*). This allows the user to define a chromatography run and is divided into five fields: I for injection, L for elution, Q for equilibration, D for data recording and F for fraction collection.

In the injection (I) field, the user defines:

(i) Which sample should be injected?

(ii) What sample volume?

(iii) On which column?

In the elution (L) field, the user defines:

(iv) Which buffer should be used for elution?

(v) At what flow rate?

(vi) Should the elution slow down when the sample is in the measurement cell to enable SANS signal recording with proper statistics?

(vii) If yes, at which flow rate and after what elution time or absorbance threshold?

(viii) How long should the slow elution phase last before it speeds up again (if the absorbance threshold has not been passed again)?

In the equilibration (Q) field, the user defines:

(ix) Should the other column be equilibrated in parallel?

(x) With which buffer and at what flow rate?

In the data recording (D) field, the user defines:

(xi) At which wavelengths should the chromatograms be recorded?

The SANS exposure is set to 30 s and repeated until one column volume is eluted, plus 10% for safety.

In the fraction collection (F) field, the user defines:

(xii) Should the sample be collected?

(xiii) Should the collection start from the first fraction position or continue the previous collection?

(xiv) With what fraction size (in time or volume)?

Several runs can be saved and executed sequentially in the launch pad. This integrated controller offers greater comfort without compromising flexibility, as most parameters can be adjusted on the fly during the execution of a predefined chromatography run using the Settings tab. Experienced SEC–SANS users can also control their experiments remotely using the recently developed remote version of *NOMAD*.

## 4. Data recovery

The chromatography parameters and results are stored in NeXus SANS data files under the ‘d22/hplc’ tab. The recorded parameters include valve positions, column and buffer names, sample injection volume, and pump flow rates at the beginning of the elution, as well as at the four user-selected wavelengths for absorbance calculation.

The full spectrum of transmitted light is available, as well as the complete absorbance spectrum throughout the elution. Additionally, the four chromatograms calculated at the user-selected wavelengths are provided. The *x* axis of these chromatograms shows time (in seconds), elution volume (in millilitres) and fraction number. The flow rate along this axis is also provided. The reduction software *GRASP* (https://www.ill.eu/users/support-labs-infrastructure/software-scientific-tools/grasp) can be used to plot scattergrams.

## 5. Performance

**Figure 4:**
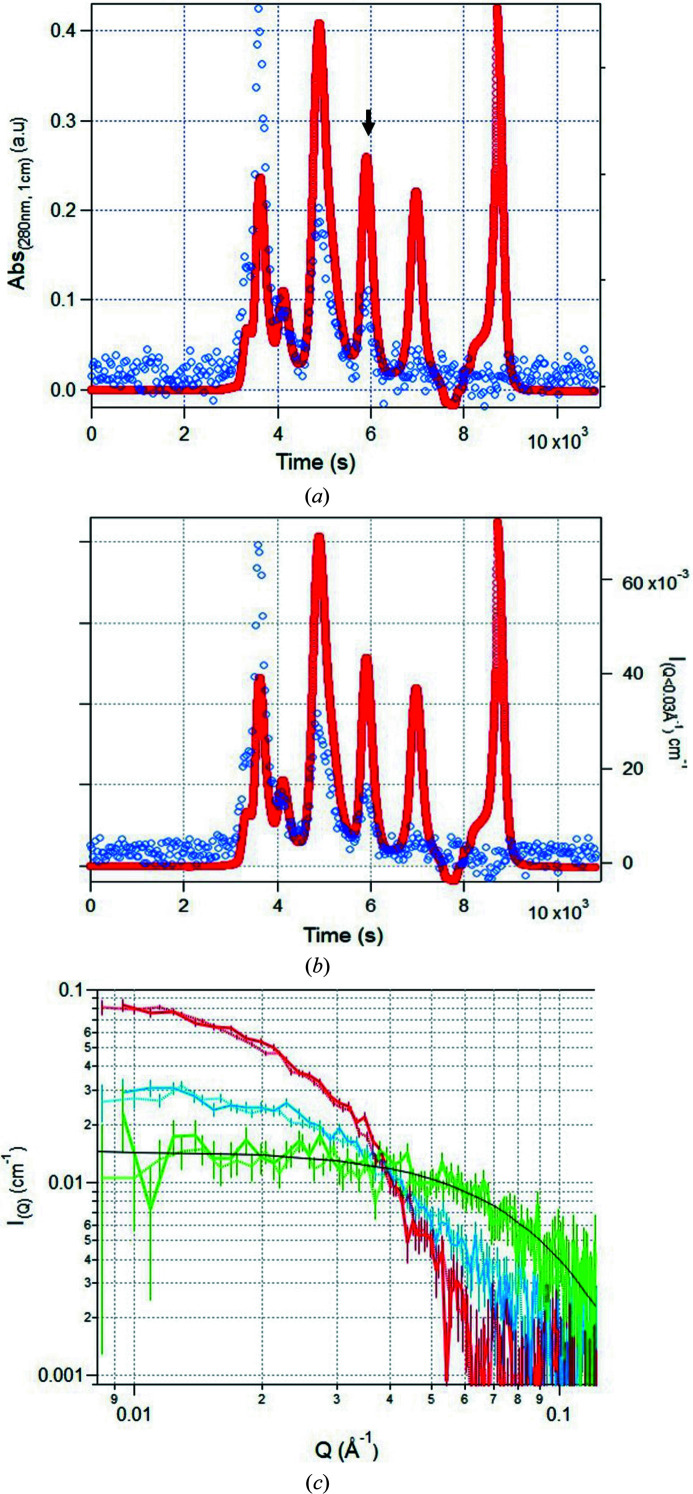
A standard protein mixture analysed using SEC–SANS. (*a*) and (*b*) Chromatograms at 280 nm (red) and scattergrams at *Q* < 0.03 Å^−1^ (blue) of 200 µl of a standard protein mix (7 mg ml^−1^) injected on a Superdex 200 (10/300) column, eluted at 0.15 ml min^−1^ and measured using a neutron wavelength of 6 Å with a spread of (*a*) 10% or (*b*) 20%. (*c*) Scattering curves extracted from the first chromatogram peak (red, thyroglobulin, 330 s exposure), the second peak (blue, γ-globulin, 120 s exposure) and the third peak [green, ovalbumin, 150 s exposure, black arrow in panel (*a*)]. Continuous lines represent data recorded using the standard 10% wavelength spread, while dotted lines represent data recorded using a 20% wavelength spread ‘high flux’ option. The continuous black line shows the theoretical curve calculated using *Pepsi-SANS* (Grudinin *et al.*, 2017 ▸) from the structure of ovalbumin (PDB ID 1ova; Stein *et al.*, 1991 ▸) at 0.35 mg ml^−1^ (42 750 kDa and ξ_280nm_ = 31 400 *M* ^−1^ cm^−1^). The data were reduced using *GRASP* and plotted using SANS reduction macros written by S. Kline (2006 ▸) for the *Igor Pro* software.

To investigate the detection limit of SEC–SANS, standard protein mixtures were measured without slowing down the flow rate when each protein reached the measurement cell. The effect of the D22 ‘high flux’ option, which relaxes the wavelength spread from 10 to 20% around the nominal wavelength, was investigated by performing two SEC–SANS runs at a neutron wavelength of 6 Å, one with 10% wavelength spread and another with 20%. The sample used was 200 µl of the SEC standard mixture reference 69385 from Sigma–Aldrich, at a total concentration of 7 mg ml^−1^, injected on an Superdex 200 10/300 column. Figs. 4 ▸(*a*) and 4 ▸(*b*) show the two chromatograms at 280 nm (in red) overlaid with the scattergrams (blue circles), which represent the averaged scattering intensity below *Q* = 0.03 Å^−1^ after subtracting the incoherent scattering, approximated by the averaged scattering intensity above *Q* = 0.13 Å^−1^. [*Q* = (4π/λ)sin(θ/2), where θ is the scattering angle and λ is the wavelength of the incident radiation.]

The two scattergrams are identical, but the reduced curves [Fig. 4 ▸(*c*)] show slightly better statistics when using a 20% wavelength spread rather than 10%, due to the increase in neutron flux. This is observed for the first three peaks, corresponding to thyroglobulin (670 kDa, shown in red), γ-globulin (150 kDa, shown in blue) and ovalbumin (43 kDa, shown in green). If we focus on the measurement of ovalbumin using a 10% wavelength spread, corresponding to the third peak in the chromatogram and the continuous green curve in Fig. 4 ▸(*c*), the concentration calculated from the forward scattering intensity [*I*(*Q* = 0) = 0.016 cm^−1^] is 0.36 mg ml^−1^ according to the *BSLDC* calculator (http://psldc.isis.rl.ac.uk/). This is in very good agreement with the concentration calculated from the light absorbance at 280 nm (Abs = 0.26), which is 0.35 mg ml^−1^ (the absorbance coefficient at 280 nm is ξ = 31 400 *M* ^−1^ cm^−1^ according to the Expasy *ProtParam* calculator, https://web.expasy.org/protparam/). This case demonstrates the low detection limit of our SEC–SANS setup, enabling us to extract a protein radius of gyration with an acquisition time of only a couple of minutes.

**Figure 5:**
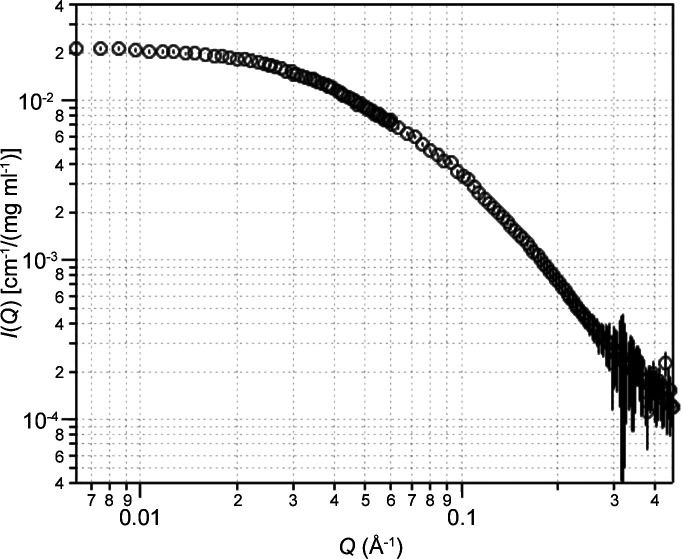
SEC–SANS data example for a 50 kDa protein–DNA complex protonated in D_2_O buffer. The complex concentration was approximately 2.5 mg ml^−1^ and the exposure time was 10 + 4 h. Data were reduced using *GRASP*, buffer subtracted, merged and concentration normalized using SANS reduction macros written by S. Kline for the *Igor Pro* software, and plotted using *Igor Pro*.

In an actual experiment, the elution flow rate is either slowed down or stopped to enable the acquisition of data with sufficient statistics for shape fitting. As an example, Fig. 5 ▸ presents data recorded from the injection of a protein–DNA complex onto a Superdex 200 10/300 Increase column (Caporaletti *et al.*, 2023 ▸). The injected sample was 200 µl at a total concentration of about 20 mg ml^−1^. Both the protein (36 321.4 Da as a dimer) and the DNA (14 845.2 Da) were protonated and the buffer was prepared with 100% D_2_O. The column diluted the sample about seven times and, according to the absorbance, the complex concentration in the SANS measurement cell was about 2.5 mg ml^−1^.

The elution flow rate (0.3 ml min^−1^) was manually stopped at the top of the chromatogram peak to record the SANS pattern over 10 h in a small-angle configuration (8 m collimation, 8 m sample-to-detector distance, 6 Å ± 10% wavelength). The instrument configuration was then changed to record the SANS pattern at a large angle (2.8 m collimation, 2 m sample-to-detector distance, 6 Å ± 10% wavelength) over 4 h. Since these data were recorded, the installation of a second detector at 1.4 m from the sample position has enabled the recording of the whole *Q* range at once. No correction factor was needed to subtract the buffer background and the data statistics were excellent, with a large-angle intensity value as low as 1 × 10^−4^ cm^−1^.

Other standard proteins measured with better statistics using the flow-rate slowing option are presented in various publications (Johansen *et al.*, 2018 ▸; Lycksell *et al.*, 2021 ▸; Johansen *et al.*, 2022 ▸; Golub *et al.*, 2022 ▸; Trewhella *et al.*, 2022 ▸).

## 6. Conclusions

The combination of SEC with SANS has the obvious dis­advantage of using more beamtime than a standard cuvette measurement. However, not only does it improve the sample quality but it also enhances the reliability of background subtraction and therefore the sensitivity of the SANS instrument. The elution runs can be prepared in advance to avoid making decisions during scarce and precious beamtime. Several runs can be programmed automatically and the samples can be recovered for further analysis. On the other hand, in the case of unforeseen sample behaviour, the instrument control interface allows for flexibility to interact with the elution while it is ongoing.

In conclusion, this setup enables the measurement of SANS data on samples of optimal quality while combining flexibility and user comfort. Given the requirement of sample monodispersity for subsequent data analysis, SEC–SANS is advised as the standard bioSANS measurement method. Interestingly, the chromatography process can be used not only for purification purposes but also for exchange. In particular, it enables the exchange of the buffer and detergent surrounding membrane proteins for their contrast-matched deuterated version [see Johansen *et al.* (2018 ▸)], saving prior sample preparation steps. This opens up the possibility of studying membrane proteins by SANS with the same efficiency as SAXS has for soluble proteins.
